# Examining individual and school-level predictors of positive attitudes towards condom use among adolescents in Tanzania: An exploratory analysis using hierarchical linear modeling

**DOI:** 10.1371/journal.pone.0324856

**Published:** 2026-11-03

**Authors:** Sara Bryk, Arisa Shichijo, Sylvia Kaaya, Isaac Lema, Anna Minja, Abby C. Cannon, Rachel Mtei, Magreat Somba, Eric van Praag, Joseph R. Egger, Joy Noel Baumgartner

**Affiliations:** 1 University of Chicago, Chicago, Illinois, United States of America; 2 University of North Carolina at Chapel Hill, Chapel Hill, North Carolina, United States of America; 3 Muhimbili University of Health and Allied Sciences, Dar es Salaam, Tanzania; 4 Iris Group, Durham, North Carolina, United States of America; 5 Independent Consultant, Dar es Salaam, Tanzania; 6 Duke University, Durham, North Carolina, United States of America; PLOS: Public Library of Science, UNITED KINGDOM OF GREAT BRITAIN AND NORTHERN IRELAND

## Abstract

**Introduction:**

Adolescents shoulder a disproportionate burden of poor sexual and reproductive health (SRH) outcomes in many lower-income countries, including Tanzania. Availability, access, and use of contraception, including condoms, reduces unintended pregnancies and sexually transmitted infections. Research indicates that early attitudes can influence future behavior, yet little is known about young adolescents’ attitudes toward condom use. This study examines whether and how knowledge, beliefs, and sociodemographic factors influence attitudes toward condom use among adolescents in Tanzania.

**Methods:**

Data are from a school-cluster randomized controlled trial with Standard 7 adolescents in two Tanzanian districts. Using an analytic sample of 1,057 adolescents from 20 primary schools, descriptive analysis and hierarchical linear modeling (HLM) were conducted to parse out individual- and school-level influences that shape attitudes towards condom use.

**Results:**

Individual and aggregate characteristics of the school environment were associated with attitudes toward condom use. At the individual level, more positive attitudes were associated with being male, having access to condoms, and having knowledge about condom use and pregnancy prevention. At the school-level, more positive attitudes were associated with a larger male population in classes, greater breadth of pregnancy knowledge among students, and having relationships with trusted adults.

**Conclusions:**

Findings suggest that adolescents largely turn to peers and adults for information and guidance regarding condoms. In turn, the views and beliefs of others can influence attitudes towards condom use. Schools might be an effective unit of change for interventions to facilitate more group-based education about the prevention of negative SRH outcomes.

## Introduction

Adolescents shoulder a disproportionate burden of poor sexual and reproductive health (SRH) outcomes in many low- and middle-income countries (LMIC) [1,2]. The incidence of sexually transmitted infections is highest among adolescents compared to other age groups [3]. Approximately half of adolescent pregnancies in LMICs are unintended, and pregnancy-related complications are the leading cause of death among adolescent girls and young women (AGYW) aged 15–19 in low-income countries. Youth and adolescents also are less likely than adults to access contraceptive services, which further contributes to these problematic outcomes [4–6].

The issues described above are pervasive in Tanzania—the context for this study [7–12]. A 2023 nationally representative survey found that HIV prevalence among females is nearly three times higher than males aged 15–19 (0.8% versus 0.3% respectively) [13]. Further, among AGYW aged 15–19, only 38% had accurate knowledge about HIV transmission and prevention methods, including the use of condoms [1]. Meanwhile, relative to other age groups, AGYW had the lowest contraceptive use rates, and the highest unmet need for family planning [2]. Among sexually active, unmarried AGYW ages 15–19, 55.2% reported an unmet need for family planning. Within this population, male condom use was the second most commonly used pregnancy prevention method (behind implants), yet only 7.4% reported current condom use [1]. Even among currently married 15–19-year-olds, there is a 22.5% unmet need for family planning. While few studies have focused on sexually active younger adolescents, the research indicates that contraceptive use rates are even lower [2,14]. Although various HIV and family planning interventions have been trialed and implemented in Tanzania over the years, these stark statistics highlight the need for further efforts to enhance appropriate uses of contraceptive methods, such as condoms for dual protection, to prevent unintended pregnancies, reduce HIV acquisition, and improve SRH outcomes, particularly among adolescents.

The literature identifies social, cultural, and demographic factors associated with adolescents’ attitudes toward and decision to use contraception. For example, past studies have found that the quality of parent-child communication about sex and sexuality appears to be a strong determinant of adolescents’ sexual behavior as well as culturally-specific parenting behaviors. Relatedly, there is increasing evidence of and interest in understanding the role of trusted adults in the lives of adolescents more broadly and how that influences adolescent health and well-being [15,16].

Other predictors of contraceptive use include contraceptive access, normative beliefs about contraception, gender, age, socioeconomic status, and religion, as well as an individual’s sense of self-efficacy, and knowledge of how someone becomes pregnant, prevents a pregnancy, and uses a condom [15,17–20]. In 2018, a scoping review by Protogerou and colleagues examined the predictors of male condom use among adolescents and adults under age 35 in sub-Saharan Africa (SSA). They found that most predictors had both direct and indirect effects on condom use. Indirect effects were mediated by “disposition to act”—known in the family planning literature as “intention to use contraception”. Importantly, across all studies in SSA and globally, the authors noted that “attitudes” was the strongest correlate of adolescent condom use [21].

Given the importance of attitudes for future condom use, it is noteworthy that few studies identify *attitudes* towards condom use as an outcome of interest. Among young adolescents who are not yet sexually active, it is difficult to examine the disposition to act and the actual use of condoms. A more robust understanding of the factors associated with early adolescent attitudes towards contraception, and condoms in particular, could help program implementers design communication programs in Tanzania (and potentially elsewhere) that educate adolescents before developing an unmet need for contraception. Importantly, by targeting these interventions at young adolescents’ pre-sexual debut, such efforts could subsequently reduce unintended pregnancies, sexually transmitted infections (STIs), the spread of HIV, and a cascade of poor health outcomes that will otherwise hinder adolescents from reaching their life goals [22].

In response to these gaps in the literature, this study explores the individual and school-level factors that shape general attitudes towards male condom use among early- and mid-adolescent students (11–17 years) in two Tanzanian districts. The objectives were:

1. *To explore attitudes towards condom use among young adolescents and whether they vary by sex.* Previous research indicates that women and men experience different norms regarding condom use. However, because much of the contraceptive research and interventions in SSA focuses on women and girls, less is known about the attitudes toward contraception among adolescent boys [14].
2. *To examine the individual- and school-level predictors of more positive attitudes towards condom use among young adolescents.* The primary predictor of interest tests the hypothesis that trusted adult relationships can foster positive attitudes towards condom use. Additional predictors, as guided by the literature, include demographic characteristics, pregnancy knowledge, and beliefs about contraceptive use, among others.

## Methods

### Study setting and study population

This current study uses baseline data from a larger parent study that aimed to assess the impact of clinic-based and school-facilitated Adolescent Wellness Visits. The parent study was a cluster randomized clinical trial registered at Clinicaltrials.gov #NCT05306938. Baseline data collection occurred between June 6 and October 3, 2022 and was collected in two sites in Tanzania: one urban district in Dar es Salaam region and one rural/peri-urban district in Coast region. A total of 1,095 students were surveyed across 20 primary schools, during their final year of primary school (Standard 7) when school attendance is typically high and when adolescents are going through or nearing puberty. Students ranged from 11 to 17 years old, with an average age of 13.4 years at baseline. Each school cluster ranged in class size from 23 to 92 students.

Ethical approvals were received from the Office of Human Research Ethics at the University of North Carolina at Chapel Hill, Muhimbili University of Health and Allied Sciences Research and Ethics Committee in Tanzania, the National Health Research Ethics Committee at the National Institute of Medical Research in Tanzania, and the Duke University Health System Institutional Review Board. Adolescents and their parents/guardians gave written informed assent and consent, respectively, prior to study participation.

### Adolescent survey data collection and management

Because our study focuses on young adolescents who are rarely sexually active, students who reported ever engaging in sexual activity (3.4%, n = 37) were removed from the analysis. This includes students who were subject to sexual violence – a group that is likely to differ from others in the sample. One participant with missing sexual activity data was also removed, resulting in a final analytic sample of 1057.

Where possible, survey items were drawn from existing sources previously tested in Tanzania and East Africa. Where new items were required, our research team developed them and subjected all items to a rigorous translation and validation process: forward and backward translation, discrepancy reports, consensus discussions, adolescent pre-testing, and final group review. All survey items were administered in Swahili.

### Construction of outcome variable

#### Attitudes towards condom use.

The survey included a series of questions framed around personal beliefs and attitudes regarding contraception, as detailed in Supplemental File Table A in S1 File. Four items assessing attitudes towards contraceptive use demonstrated acceptable internal consistency (Cronbach’s Alpha = 0.74) and were averaged to form the outcome variable “Using a condom is a way of expressing responsibility for my partner and myself”, “It is okay for a girl to suggest condom use to a sexual partner”, “It is okay for girls my age to carry condoms if they plan to have sex”, and “It is okay for boys my age to carry condoms if they plan to have sex.” Each item was scaled from 1 to 5, with higher scores reflecting more positive attitudes. These items were drawn from previous surveys used to assess the effect of school-based HIV prevention programs on adolescent sexual behavior in SSA where the research found that talking about condoms with adolescents does not negate an intervention’s ability to both delay sex and increase condom use [23].

*Construction of key predictors (details in* Table 1A*).*

**Table 1:** Sex-disaggregated characteristics of adolescents, age 11-17, in Dar es Salaam and Coast regions, surveyed in 2022 in Tanzania (N = 1,057).

Characteristics	Overall (N) (%) N = 1,057	Female (N) (%) N = 566	Male (N) (%) N = 491
Reached puberty	632 (59.8%)	286 (50.5%)	346 (70.5%)
Socioeconomic Status (National Quintile) (n = 932)			
Quintile 1 (lowest wealth)	5 (0.5%)	2 (0.4%)	3 (0.7%)
Quintile 2	42 (4.5%)	24 (4.8%)	18 (4.2%)
Quintile 3	52 (5.6%)	38 (7.5%)	14 (3.3%)
Quintile 4	211 (22.6%)	124 (24.6%)	87 (20.4%)
Quintile 5 (highest wealth)	622 (66.7%)	317 (62.8%)	305 (71.4%)
Religion (n = 1056)			
Christian	343 (32.5%)	176 (31.2%)	167 (34.1%)
Muslim	713 (67.5%)	390 (68.8%)	323 (65.9%)
**Characteristics**	**Overall** Mean (SD)	**Female** Mean (SD)	**Male** Mean (SD)
Age	13.4 (1.2)	13.2 (1.2)	13.6 (1.2)
Socioeconomic Status (National Score) (n = 1056)	0.8 (0.5)	0.7 (0.5)	0.9 (0.5)
General self-efficacy score	29.4 (6.9)	28.9 (6.9)	30.0 (6.8)
Trusted adult relationship (n = 1037) • If needed, do you have a trusted adult who could help you access contraception now or in the future?	0.7 (0.5)	0.7 (0.5)	0.6 (0.5)
Pregnancy knowledge (2 items combined below)	0.8 (0.4)	0.7 (0.4)	0.8 (0.3)
1) Do you know how people get pregnant?	0.8 (0.4)	0.8 (0.4)	0.9 (0.3)
2) Do you know any methods of preventing pregnancy?	0.7 (0.5)	0.7 (0.5)	0.7 (0.5)
Condom access (2 items combined below) (n = 1053)	0.4 (0.4)	0.3 (0.4)	0.5 (0.4)
1) Do you know of a place where an adolescent can get condoms? (n = 1055)	0.5 (0.5)	0.4 (0.5)	0.6 (0.5)
2) If you wanted to, could you get yourself a condom? (n = 1053)	0.3 (0.4)	0.2 (0.4)	0.3 (0.5)
**For the following questions, students were asked to select the response that best reflects their beliefs from strongly disagree (1) to strongly agree (5).** Higher scores (5) represent more positive beliefs regarding contraception (disagreement with the stated items).			
Attitudes towards condoms (average of the following 4 items below):	3.2 (0.9)	3.1 (1.0)	3.4 (0.9)
1) Using a condom is a way of expressing responsibility for my partner and myself.	3.2 (1.2)	3.0 (1.2)	3.4 (1.2)
2) It is okay for a girl to suggest condom use to a sexual partner.	3.4 (1.2)	3.2 (1.2)	3.6 (1.1)
3) It is okay for girls my age to carry condoms if they plan to have sex.	3.1 (1.3)	2.9 (1.3)	3.3 (1.3)
4) It is okay for boys my age to carry condoms if they plan to have sex.	3.2 (1.3)	3.1 (1.3)	3.4 (1.3)
Condom use knowledge I am able to use a condom correctly	2.6 (1.4)	2.5 (1.4)	2.8 (1.5)
**For the following questions, students were asked to select the response that best reflects their beliefs from strongly agree (1) to strongly disagree (5).** Higher scores (5) represent more positive beliefs regarding contraception (disagreement with the stated items).			
Parental views on contraception • My parents would punish me if they found me with contraceptives.	3.1 (1.4)	3.0 (1.4)	3.2 (1.4)
Beliefs about contraceptive use (2 items combined below)	3.7 (1.0)	3.7 (1.0)	3.8 (1.0)
1) Contraception is only for adult married persons.	3.9 (1.2)	3.9 (1.1)	3.9 (1.2)
2) Adolescents who use contraceptives are considered bad.	3.6 (1.3)	3.5 (1.3)	3.6 (1.3)

Demographic predictors included sex (male or female), age (in years), puberty status (pre-puberty or puberty reached), and religion (Christian or Muslim).

#### Socioeconomic status (SES).

Adapted from the Demographic and Health Survey, a socioeconomic indicator was constructed using the Tanzanian Equity Tool [24]. This tool assigns a continuous score based on items owned, building materials used in the house, and main energy source, among other factors. When estimating a continuous national score, one question regarding bank accounts was omitted from the measure because over 10% of students were unsure how to answer.

#### The general self-efficacy scale (GSE).

The GSE was calculated by summarizing responses from ten survey items related to knowledge and confidence in one’s ability to problem solve and persevere through difficult situations [25].

#### Trusted adult (primary predictor).

Students were asked whether they had a relationship with a trusted adult who could help them access contraception now or in the future. This question was derived from a scoping review protocol, designed to examine the relationship between trusted adults and adolescent outcomes [16].

#### Parental views on contraception.

Students were asked whether they believed their parents would punish them if they were found with contraception.

#### Beliefs about contraceptive use.

The belief that “contraception is only for adult married persons” and that “adolescents who use contraceptives are considered bad” were moderately correlated (r = 0.40). These items were averaged into a single measure reflecting contraceptive beliefs.

#### Pregnancy knowledge.

Knowing how a person becomes pregnant and knowing at least one method of preventing pregnancy were correlated (r = 0.62). Per the skip logic in the survey, the latter item (knowing a prevention method) was recoded as a 0, if the student answered no to the former item (knowing how a person becomes pregnant). These binary items were averaged into a single measure reflecting pregnancy knowledge.

#### Condom use knowledge.

Students were asked whether they knew how to use a condom correctly.

#### Condom access.

A correlation of 0.62 indicates that two items related to condom access (knowing where to get condoms and believing in one’s ability to get a condom, if needed) form a cohesive measure. Per the skip logic in the survey, the latter item (believing in one’s ability) was recoded as a 0, if the student answered no to the former item (knowing where to get condoms). We averaged these two items together to generate a continuous predictor, where a high score reflects high levels of confidence and knowledge about how to access condoms.

### Statistical modeling

Descriptive statistics were used to summarize participant characteristics. Hierarchical linear modelling (HLM) was used to examine whether and how various factors are associated with early adolescent attitudes toward condom use. HLM makes it possible to simultaneously parse out the individual- and school-level (i.e., contextual) influences that shape adolescent attitudes. This is important, given that previous studies note the limitations of single-level models [26]. For example, Protogerou and colleagues found that individual-level social-cognitive determinants accounted for a small percentage of the variance in adolescent condom use intentions and behaviors [21]. Other researchers hypothesized that site- or school-level variables may play a significant role and called for interventions and analyses to include interpersonal and structural determinants [17,26]. We note that prior to HLM, we examined the correlation matrices and did not see any evidence of multicollinearity or problematic variance inflation factors (VIFs)**.** None of the correlations among the level 1 predictors exceeded 0.5 and the VIF did not exceed 1.45.

With each model, predictors were added in a stepwise fashion, beginning with the background characteristics. HLM was used to simultaneously estimate both the student-level (level-1) and school-level (level 2) effects. From calculating the intraclass correlation of the unconditional model, we found that 4.9% of the total variance in adolescent attitudes was due to differences between schools (Table 2, Model 1). To determine whether school-level variation persisted after controlling for student characteristics, a random intercepts HLM model was run with all student-level predictors grand mean centered (Table 2, Model 2). Significant residual variation continued to exist at the school-level. A likelihood ratio test comparing a model where intercepts vary across schools versus a restricted fixed intercept model was statistically significant [26]. With evidence of significant outcome differences among contexts, we explored possible predictors of this variation.

**Table 2:** Results of the hierarchical linear models, with individual characteristics added in a stepwise fashion, and school-level predictors entered individually (N = 1,057 students, 20 schools).

Variable	Estimate (SE)				
	*Fixed effects*				
	Student level predictors				
	Model 1: Unconditional model	Model 2: Grand-mean centered model	Model 3: Grand mean centered model with proportion of female students included at school-level	Model 4: Grand mean centered model with trusted adult included at school-level	Model 5: Grand mean centered model with pregnancy knowledge included at school-level
Intercept	2.23*** (0.06)	3.23*** (0.03)	3.89*** (0.18)	2.85*** (0.17)	2.77*** (0.19)
Sex (female)		−0.18*** (0.05)	−0.16*** (0.06)	−0.19*** (0.05)	−0.18*** (0.05)
Age		0.01 (0.02)	0.01 (0.02)	0.01 (0.02)	0.01 (0.02)
Puberty reached		−0.05 (0.06)	−0.05 (0.06)	−0.04 (0.06)	−0.04 (0.06)
Socio-economic status score		0.01 (0.06)	−0.01 (0.05)	−0.03 (0.06)	−0.04 (0.06)
Religion (Christian)		−0.06 (0.06)	−0.05 (0.06)	−0.07 (0.06)	−0.07 (0.06)
Self-efficacy score (GSE)		0.01 (0.00)	0.01* (0.00)	0.00 (0.00)	0.00 (0.00)
Trusted adult		−0.07 (0.06)	−0.06 (0.06)	−0.09 (0.06)	−0.08 (0.06)
Parental views on contraception		0.02 (0.02)	0.02 (0.02)	0.02 (0.02)	0.02 (0.02)
Beliefs about contraceptive use		−0.03 (0.03)	−0.03 (0.03)	−0.03 (0.03)	−0.03 (0.03)
Pregnancy knowledge		0.56*** (0.08)	0.56*** (0.08)	0.53*** (0.08)	0.52*** (0.08)
Condom use knowledge		0.17*** (0.02)	0.16*** (0.02)	0.17*** (0.02)	0.17*** (0.02)
Access to condoms		0.56*** (0.07)	0.56*** (0.07)	0.56*** (0.07)	0.55*** (0.07)
	**School-level predictors**				
Proportion of female students			−1.23*** (0.34)		
Proportion of students with trusted adult relationships				0.57** (0.25)	
Average pregnancy knowledge in schools					0.60** (0.26)
***Random effects***	***Variance component***				
Intercept, 𝜇_0j_	0.04411	0.00963	0.00032	0.00569	0.00471
Student level effect, r_ij_	0.86418	0.68634	0.68598	0.68609	0.68650
***Model Fit***					
ICC	4.9%				
Deviance	2808.82	2604.10	2591.84	2598.47	2601.46

***Significance at alpha level of 0.01. **Significance at alpha level of 0.05. *Significance at alpha level of 0.1.

To investigate possible school-level effects, mean aggregates of all 12 individual-level variables were considered for inclusion at level 2. Analyses were run one variable at a time to explore potential school-level compositional effects (Table 2, Models 3−5). More complex level-2 models were not feasible given that the data set was limited to 20 schools. Only models with significant level-2 variables were included in our results (using a predefined alpha threshold of 0.05)*.*

When level-1 predictors are grand-mean centered and their corresponding school mean aggregates are included as level-2 predictors, the intercept represents the adjusted mean outcome for each site. This approach enables us to model the differences between schools, while controlling for the composition of students within schools. In other words, the level-2 predictors estimate the extent to which aspects of the school environment affect the outcome above and beyond the effects of individual differences within schools on these same factors [26].

See Supplemental file B in S1 File for the notation of the final model. All data were first imported into STATA (version 18; StataCorp), where it was cleaned. All models were then run in HLM Version 8 Scientific Software International, Inc. software.

## Results

Our analytic sample included 1,057 adolescents (566 female; 491 male). Their demographic characteristics and sexual health knowledge and attitudes are summarized in Table 1, disaggregated by sex. Students’ average age was 13.4 years and the sample was primarily Muslim (68%), female (54%), and had reached puberty (60%). The majority (85%) of students said they were knowledgeable about how people became pregnant, and 68% knew how to prevent a pregnancy. Half (50%) of students knew where they could access condoms or other contraception, while only 28% felt confident about their ability to obtain condoms for themselves. A substantial proportion (66%) of students had a relationship with an adult who they trusted to help them access contraception if needed. When students were asked about their attitudes and beliefs towards condoms and contraception, with few exceptions, the average response was typically neutral (neither agree nor disagree with the proposed statement). Of note, the majority disagreed with the statement that contraception was only for adult married persons. On average, students lacked confidence in their ability to use a condom correctly. Students had differing beliefs regarding how their parents would react to finding them with contraception; 45% agreed with the statement that their parents would punish them, 50% disagreed, and the remainder were neutral. No specific pattern by sex was observed.

The results of the HLM are detailed in Table 2. Four individual (level-1) effects that met our predefined threshold of statistical significance (defined as p < 0.05) were identified: student sex, pregnancy knowledge, condom use knowledge and students’ ability to access condoms (Model 2). Three school-level (level-2) effects that met our statistical significance threshold were identified: the sex composition of the school (Model 3); the presence of trusted adult relationships (Model 4); and the breadth of pregnancy knowledge among students (Model 5). At the school-level, beliefs about contraceptive use, parental views on contraception, and access to condoms, approached but did not meet our predefined significance threshold. There was no evidence of contextual effects among the other predictors. Additional sensitivity analyses using ordinary least squares and fixed effect models were run and confirmed similar findings (Supplemental File Table A2 in S1 File). The significant effects are summarized as follows.

### Sex

*Adolescent* girls had more negative attitudes towards condom use than boys, and this effect was amplified at the school level (p < 0.001). In schools with a higher proportion of girls, average attitudes towards contraception were more negative, a finding that was beyond the summative effect of simply having more girls with more negative attitudes (p < 0.001).

### Trusted adult (primary predictor of interest)

Having a relationship with a trusted adult who could help a student access contraception was not a significant predictor of individual attitudes towards condoms. Interestingly however, in schools where more students had trusted relationships with adults, their average attitude towards condom use was also more positive, signaling a potential school effect (p = 0.037).

### Pregnancy knowledge

Students who had more knowledge about how a person gets pregnant and how to prevent pregnancy had more positive attitudes toward condoms, compared to students who lacked this knowledge (p < 0.001). When average pregnancy knowledge in the school was higher, attitudes towards condoms were more positive (p = 0.020).

### Condom use knowledge

Students who knew how to use a condom had more positive attitudes towards condoms as compared to those who did not (p < 0.001).

*Access to condoms*

At the individual level, when students had high levels of confidence and knowledge about where and how to access condoms, their attitudes toward condoms were more positive (p < 0.001).

## Discussion

Global evidence indicates that increased condom use among adolescents can prevent unintended pregnancy, STIs/HIV, and associated adverse health outcomes. It can also improve opportunities for educational and economic advancement. However, globally and in Tanzania, the rate of condom use among adolescents remains low, and HIV knowledge, attitudes, and behaviors are highly variable. While various SRH interventions have been trialed in Tanzania, the majority focused on older adolescent girls and young women. Researchers have called for interventions targeted towards early adolescents before gendered norms are deeply ingrained and unsafe sex practices have begun [27–29]. With that hypothesis in mind, we conducted this exploratory analysis to better understand the individual and aggregate characteristics of the school environment that shape young adolescents’ attitudes towards condoms.

Regarding gender, our study found that adolescent girls had more negative attitudes towards condoms than boys, and this effect was amplified at the school level. We wonder if the differential norms faced by girls and boys may contribute to the negative attitudes observed amongst girls. In Mozambique, researchers found that young women who used condoms were perceived as not “decent,” and thought to be having sex with multiple partners. In contrast, young men were more encouraged to be “sexually free” [30]. Similarly, a study on contraceptive decision making in Kenya, Nigeria, and Uganda found that girls more frequently reported hiding their contraceptive use from parents and partners to avoid stigma that boys were less subject to [31]. While it seems cultural and social norms likely play a part in shaping attitudes among young adolescents, this remains an area for further investigation. Importantly, research also suggests that boys and men have more decision-making influence regarding contraceptives, particularly in contexts where girls have less autonomy and choice over their SRH decisions [27]. Including boys in SRH interventions is critical, especially given their relatively positive attitudes towards condom use.

Numerous studies also have found that barriers to contraceptive access are associated with decreased contraception use [21]. This study finds that lacking knowledge about where and how to get condoms was associated with more negative attitudes towards its use. Aventin et al. found that adolescents in Southern African countries often have limited or inaccurate information about pregnancy and contraceptive use [18]. Consistent with our findings, studies also have shown that when adolescents feel knowledgeable about pregnancy and condom use, their attitudes towards condoms are more positive [18,19]. These three characteristics – pregnancy knowledge, access to contraceptive methods, and knowing how to use a condom – all suggest that increased sexual and reproductive health knowledge may positively influence students’ attitudes towards condoms.

At the school level, positive attitudes towards condoms were amplified when the student body was predominantly male, when students were better informed about pregnancy and its prevention, and when a larger proportion of students had trusted adult relationships (Table 2, Models 3, 4, and 5). These contextual effects align with previous research showing that peers turn to one another as confidants when seeking information regarding contraception and can even act as facilitators of condom use by sharing condoms with their friends [20]. Conversely, in social groups where negative norms were prevalent, these norms acted as a barrier to use. Such studies align with Aventin and colleagues’ finding that peer norms can act as a positive or negative influence on condom use [20].

The effect of having a trusted adult relationship appears more complex than anticipated. The individual and composition effects appear to work in opposite directions, with the relationship having a nonsignificant negative effect on attitudes at the individual level, and a significant positive effect at the school-level. However, given that the individual level effects are nonsignificant across all the models, we cannot meaningfully interpret these results. The school-level effect suggests that students may be responsive to the attitudes of teachers or other trusted adults in their community (Table 2, Model 4). We hypothesize that teachers who have and share a positive outlook toward contraception could positively influence their students’ perspectives. In future research, it would be helpful to better understand the breadth and depth of those social connections, and how that relationship may shape adolescent attitudes.

### Limitations

First, given that this analysis was conducted in a limited number of schools and districts, the findings are not widely generalizable. Future research would benefit from larger school samples to study these phenomena in greater detail, and to have a more robust basis for inference. Additionally, given that this was a school-based intervention, generalization to the greater population of early- and mid-adolescents (including out-of-school youth) is not possible. Previous research has found schooling to be a protective factor against sexual risks. Modern contraceptive use is also found to increase with increasing education, and so we suspect that out-of-school youth may have a different experience [1]*.* Second, past research has identified additional predictors of adolescent attitudes toward condom use that were not captured in this dataset. While these omitted variables could be a source of bias to the model, we addressed these concerns by including proxy variables. For example, the Demographic and Health Surveys in Tanzania and other neighboring countries have shown that condom use varies by living conditions (e.g., urban, peri-urban, and rural) [1]. While precise living classifications for students were unavailable, a socio-economic measure was included as a proxy. Differences in home environments also are likely to be picked up by the inclusion of school-level variables in these models.

Several of our predictors (e.g., beliefs about contraceptive use, pregnancy knowledge, and condom access) are two-item scales, averaged from binary variables, which may bias our analysis towards a null result. While we recognize that multi-item scales may have higher internal reliability and consistency, we still found that the latter two predictors were internally correlated and statistically significant, indicating that these scales are likely valid and also tap an underlying latent construct. Future researchers may see even stronger findings, if expanded scales are used.

Certain model assumptions were required to conduct the analyses. The level-2 model assumes normally distributed random effects. No evidence of a serious violation of the assumptions was seen in a QQ plot of the residuals. The linearity assumption was assessed via scatterplots of each predictor against the outcome and no violations were detected. Given the small number of schools in the sample, there could also be a concern about unreliable standard errors. It is important to note the effect sizes for the three level-2 predictors are several times larger than the standard error. Even if the standard errors were underestimated, it is unlikely to significantly change the statistical inferences. Further, these probabilities were estimated using approximate degrees of freedom, which account for potential unreliability in the standard errors. HLM also assumes that errors at levels 1 and 2 are not correlated. Given that the level-1 estimates did not vary drastically after inclusion of the level-2 predictors, we are not concerned about cross-level correlated errors (Table 2). Lastly, as a sensitivity analysis, ordinary least squares and fixed effect models were run and compared to the group- and grand-mean centered models at level 1 (Supplemental File Table A2 in S1 File). The coefficients and standard errors are similar across all four models. This is further evidence of a lack of confounding due to cross-level errors.

### Program and policy implications

Our findings have programmatic and policy implications. If we aim to promote positive attitudes towards condom use among young adolescents to influence future protective behaviors (preventing unwanted pregnancies and STIs/HIV), then the school, particularly female-dominated schools, might be an important unit of change for effective interventions. Across the globe, schools have been used as an interventional setting for SRH programs – many of which aim to change attitudes, intentions, behaviors, and social norms by improving students’ knowledge, understanding, and skills. School interventions have taken various forms, including delivery by teachers or peer educators, providing incentives for staying in school, imparting knowledge and skills to delay sexual debut, provision of free contraceptives, and facilitated connections to health service providers, among others. Just as the intervention designs vary, so do the intended outcomes and results [32]. For example, one systematic review found that school- and community-based interventions improved adolescent knowledge, attitudes, and/or SRH practices [33]. In contrast, a Cochrane review of six school-based educational interventions found no demonstrable effects on the prevalence of HIV, STIs, or pregnancy. The authors hypothesize that these interventions may be more effective if coupled with other elements, such as contraceptive provision, and poverty reduction efforts [32].

Given that positive attitudes towards condom use were associated with increased knowledge, providing adolescents with more skills-based information regarding safer sex practices (e.g., healthy communication in romantic relationships) -- coupled with information about where and how to use and access contraceptives -- may increase their likelihood of developing positive attitudes towards condom use in the future. Doing so in a school setting might then allow students to share and reflect on that knowledge together and with trusted adults. Additional research could test these hypotheses, and whether early positive attitudes towards condom use subsequently translates into future use of condoms, as well as a reduction in HIV, STIs, unintended pregnancies, and associated adverse health outcomes.

## Conclusion

This study provides important insights into the individual and school-level factors that shape young adolescents’ attitudes towards condom use in Tanzania. After adjusting for other covariates, adolescent girls demonstrated more negative attitudes towards condoms than boys, potentially reflecting the disproportionate stigma they face around contraceptive use — a pattern consistent with broader evidence from sub-Saharan Africa. The relatively positive attitudes observed among adolescent boys highlight the critical importance of engaging them as active participants in SRH programming.

Our results also emphasize that knowledge is a powerful driver of attitudes: adolescents who understood pregnancy, knew how to use condoms, and knew where to access them held more favorable views toward condom use. This suggests that comprehensive, age-appropriate SRH education may help reduce unsafe sexual behavior. Trusted adult relationships further shaped these attitudes at the school level, pointing to the broader social environment as a meaningful lever for change.

## Supporting information

S1 Data Dataset. (XLS)

S1 File Supplemental tables and appendices. (DOCX)
