# Supplementary material for: Examining individual and school-level predictors of positive attitudes towards condom use among adolescents in Tanzania: An exploratory analysis using hierarchical linear modeling
Source: PLoS One. 2026 Nov 3;21(11):e0324856. doi: 10.1371/journal.pone.0324856 (PMC13730346; doi:10.1371/journal.pone.0324856)
Supplement: Supplementary file 1 [file pone.0324856.s002.docx]

**Supplemental Files**

**Table A1.** Variable construction.

| **Demographic predictors** | | |
| --- | --- | --- |
| **Construct name** | **Notes** | |
| Sex | 1 female; 0 male | |
| Age (years) |  | |
| Reached puberty | 1 puberty reached; 0 not yet reached puberty | |
| Socioeconomic Status  (Equity Tool) | National Score created from the following items:   - Does your household have electricity? - Does your household have a television? - Does your household have a radio? - Does your household have an iron? - What is the main material of the floor of your dwelling? - What is the main material of the exterior walls of your dwelling? - What is the main material of the roof of your dwelling? - What type of fuel does your household mainly use for cooking? - What is the main source of energy for lighting in the household?   1 yes; 0 no | |
| Religion | 1 Christian; 0 Muslim | |
| **Construct name** | **Survey items** | **Notes** |
| Pregnancy knowledge (0 to 1) | Average of the following two binary survey items:   - Do you know how people get pregnant? - Do you know any methods of preventing pregnancy? | r = 0.62  1 yes; 0 no |
| Condom access (0 to 1) | Factor score of the following two binary items:   - Do you know of a place where an adolescent can get condoms? - If you wanted to, could you get yourself a condom?   Average of the following two binary items:   - Do you know of a place where an adolescent can get condoms? - If you wanted to, could you get yourself a condom? | r = 0.62  1 yes; 0 no |
| Trusted adult relationship (binary) | If needed, do you have a trusted adult who could help you access contraception now or in the future? | 1 yes; 0 no |
| **For the following questions, students were asked to select the response that best reflects their beliefs from strongly disagree (1) to strongly agree (5).** | | |
| ***Primary Outcome***  Attitudes towards condoms  (1 to 5) | Average of the following items:   - Using a condom is a way of expressing responsibility for my partner and myself. - It is okay for a girl to suggest condom use to a sexual partner. - It is okay for girls my age to carry condoms if they plan to have sex. - It is okay for boys my age to carry condoms if they plan to have sex. | Higher scores (5) reflect more positive views on condom usage.  1 strongly disagree; 2 disagree; 3 neutral; 4 agree; 5 strongly agree  Cronbach's Alpha = 0.74 |
| Condom use knowledge  (1 to 5) | I am able to use a condom correctly | Higher scores (5) represent students’ higher belief that they can use a condom correctly.  1 strongly disagree; 2 disagree; 3 neutral; 4 agree; 5 strongly agree |
| Beliefs about contraceptive use (1 to 5) | Average of the following items:   - Contraception is only for adult married persons. - Adolescents who use contraceptives are considered bad. | Higher scores (5) represent more positive beliefs regarding contraception (disagreement with the stated items).  1 strongly agree; 2 agree; 3 neutral; 4 disagree; 5 strongly disagree  ( r = 0.40). |
| Parental views on contraception (1 to 5) | My parents would punish me if they found me with contraceptives. | Higher scores (5) represent more positive parental views about contraceptive use (disagreement with the stated items).  1 strongly agree; 2 agree; 3 neutral; 4 disagree; 5 strongly disagree |
| General self-efficacy scale  (10 to 40) | Sum of the following survey items:   - I can always manage to solve difficult problems if I try hard enough - If someone opposes me, I can find the means and ways to get what I want. - It is easy for me to stick to my aims and accomplish my goals. - I am confident that I could deal efficiently with unexpected events. - Thanks to my resourcefulness, I know how to handle unforeseen situations. - I can solve most problems if I invest the necessary effort. - I can remain calm when facing difficulties because I can rely on my coping abilities. - When I am confronted with a problem, I can usually find several solutions. - If I am in trouble, I can usually think of a solution - I can usually handle whatever comes my way. | Higher score indicates greater self-efficacy 1 not true at all; 2 hardly true; 3 moderately true; 4 exactly true |

**Table A2.** Ordinary least squares and fixed effect models were run as a sensitivity analysis.

|  | **Estimate (SE)** | | |  |  |
| --- | --- | --- | --- | --- | --- |
|  | Model 1A: OLS model | Model 2A: FE model, with standard errors adjusted for heteroskedasticity and clustering | | Model 3A: Group-mean centered model | Model 4A: Grand-mean centered model |
| Intercept | 1.99*** (0.36) | 2.31*** (0.48) | | 3.22*** (0.06) | 3.23*** (0.03) |
| Sex (female) | -0.19*** (0.05) | -0.16*** (0.05) | | -0.16*** (0.06) | -0.18*** (0.05) |
| Age | 0.01 (0.02) | -0.00 (0.03) | | -0.00 (0.03) | 0.01 (0.02) |
| Puberty reached | -0.06 (0.06) | -0.04 (0.06) | | -0.04 (0.06) | -0.05 (0.06) |
| SES | 0.02 (0.05) | -0.01 (0.05) | | -0.01 (0.06) | 0.01 (0.06) |
| Religion (Christian) | -0.06 (0.06) | -0.06 (0.08) | | -0.06 (0.06) | -0.06 (0.06) |
| Self-efficacy score (GSE) | 0.01* (0.00) | 0.00 (0.01) | | 0.00 (0.00) | 0.01 (0.00) |
| Trusted adult | -0.06 (0.06) | -0.09 (0.06) | | -0.09 (0.06) | -0.07 (0.06) |
| Parental views on contraception | 0.02 (0.02) | 0.01 (0.03) | 0.01 (0.02) | | 0.02 (0.02) |
| Beliefs about contraceptive use | -0.03 (0.03) | -0.03 (0.04) | -0.03 (0.03) | | -0.03 (0.03) |
| Pregnancy knowledge | 0.58*** (0.08) | 0.52*** (0.09) | 0.52*** (0.08) | | 0.56*** (0.08) |
| Condom use knowledge | 0.16*** (0.02) | 0.17*** (0.03) | 0.17*** (0.02) | | 0.17*** (0.02) |
| Condom access | 0.56*** (0.07) | 0.55*** (0.07) | 0.55*** (0.07) | | 0.56*** (0.07) |

***Significance at alpha level of 0.01. **Significance at alpha level of 0.05. *Significance at alpha level of 0.1

**Appendix B.**

**Model 1.** Hierarchical linear model, with individual characteristics added in a stepwise fashion, and school-level predictors entered individually.

Student characteristics (level-1):

Y_ij_ = β_0j_ + β_1j_(X_1ij_ - x̄_1_..) + β_2j_(X_2ij_ - x̄_2_..) + β_3j_(X_3ij_ - x̄_3_..) + β_4j_(X_4ij_ - x̄_4_..) + β_5j_(X_5ij_ - x̄_5_..) + β_6j_(X_6ij_ - x̄_6_..) + β_7j_(X_7ij_ - x̄_7_..) + β_8j_(X_8ij_ - x̄_8_..) + β_9j_(X_9ij_ - x̄_9_..) + β_10j_(X_10ij_ - x̄_10_..) + β_11j_(X_11ij_ - x̄_11_..) + β_12j_(X_12ij_ - x̄_12_..) + r_ij_

School characteristics (level 2):

β_0j_ = ɣ_00_ + ɣ_01_x̄_1j_ + ɣ_07_x̄_7j_ + ɣ_10_x̄_10j_ + u_0j_

β_1j =_ ɣ_10_

β_2j_ = ɣ_20_

β_3j_ = ɣ_30_

β_4j_ = ɣ_40_

β_5j_ = ɣ_50_

β_6j_ = ɣ_60_

β_7j_ = ɣ_70_

β_8j_ = ɣ_80_

β_9j_ = ɣ_90_

β_10j_ = ɣ_100_

β_11j_ = ɣ_110_

β_12j_ = ɣ_120_

*Where*

*X_1_* is student sex;

*X_2_* is student SES*;*

*X_3_* is student religion;

*X_4_* is student age;

*X_5_* is student puberty status;

*X_6_* is student GSE score

*X_7_* indicates whether the student has a trusted adult who could help them access contraception;

*X_8_* is parental views on contraception;

*X_9_* is student beliefs about condom use;

*X_10_* indicates whether the student is knowledgeable of how a person becomes pregnant, and how to prevent a pregnancy;

*X_11_* indicates whether the student knows how to use a condom correctly;

*X_12_* indicates whether the student has the knowledge and confidence in their own ability to access condoms;
